# Bridging the Gap: Evaluation of an Electrocardiogram Curriculum for Advanced Practice Clinicians

**DOI:** 10.5811/westjem.18085

**Published:** 2024-02-09

**Authors:** Steven Lindsey, Tim P. Moran, Meredith A. Stauch, Alexis L. Lynch, Kristen Grabow Moore

**Affiliations:** * Emory University, Department of Emergency Medicine, Atlanta, Georgia; † Emory University School of Medicine, Atlanta, Georgia

## Abstract

**Background:**

Training programs for advanced practice providers (APP) often have significant variability in their curriculum, including electrocardiogram (ECG) education. Despite limitations in formal ECG training, APPs in the emergency department (ED) may be the first practitioner to interpret an ECG. Foundations of Emergency Medicine (FoEM) offers free, open-access curricula that are widely used for resident education. We sought to improve APP ECG interpretation skills by implementing the FoEM ECG I course.

**Methods:**

This was a single-site, pre- and post-intervention study of 23 APPs at our high-acuity, urban ED. In the fall of 2020, APP learners enrolled in a FoEM ECG I course led by faculty and senior resident instructors. The course consisted of six virtual, small-group, active-learning ECG workshops. Participants completed a 15-question multiple-choice test before, immediately after, and six months post-intervention to quantify knowledge acquisition. Additionally, a pre- and post-intervention knowledge, attitudes, and practices survey was administered on ECG interpretation skills and to evaluate the course. We evaluated change in ECG knowledge scores using a Wilcoxon signed-rank test. Changes in self-assessed knowledge were evaluated using an ordinal logistic mixed-effects regression.

**Results:**

A total of 23 APPs enrolled in the course. Knowledge assessments showed APPs improved from the pre-test (median 9/15, interquartile range [IQR] 7–11) to the post-test (median 12/15, IQR 10–13; *P* = 0.001). Test scores did not significantly change from the post-test to the delayed post-test (median 12/15, IQR 12–13; *P* = 0.30). Respondents’ subjective rating of their skill did not significantly change (*P* = 0.06). Respondents reported no change in their likelihood of approaching an attending when uncertain of the correct interpretation of an ECG (*P* = 0.16). Overall, 91% were satisfied with the course and 96% agreed that the course difficulty was appropriate.

**Conclusion:**

The FoEM ECG course provided a standardized curriculum that improved APP knowledge for interpreting ECGs. Despite this, the course did not alter APPs’ willingness to approach physicians for guidance with interpretation of abnormal ECGs. These findings may inform expansion of this concept for other programs who desire formalized APP ECG education.

Population Health Research Capsule What do we already know about this issue?

*Advanced practice providers (APP) are responsible for seeing a significant number of patients in many ED settings, yet their in-training and post-training curricula are variable.*

What was the research question?

*Would the implementation of the Foundations of Emergency Medicine (FoEM) ECG I course improve electrocardiogram interpretation skills of APPs?*

What was the major finding of the study?

*Knowledge assessments improved from the pre-test (median 9/15, IQR 7–11) to the post-test (median 12/15, IQR 10–13; P = 0.001).*

How does this improve population health?

*Use of the FoEM ECG I curriculum for APP learners led to an improvement in ECG knowledge, while maintaining attending physician guidance in the setting of uncertainty.*

## INTRODUCTION

Advanced practice providers (APP), comprising physician assistants (PAs) and nurse practitioners (NPs), have a substantial presence in emergency departments (ED) in the United States. Emergency departments employed 77% of APPs in 2006, increasing from 28% in 1997.^1^ There are over 13,000 PAs and over 10,000 NPs currently practicing in the acute care setting.^2^ ^,^ ^3^ It is estimated that APPs see 21% of all ED visits and the proportion of high acuity services independently billed by APPs is increasing.^4^ ^,^ ^5^ Some models of ED care, such as practitioner-in-triage, often employ APPs as the first point of contact for patients and are tasked with ordering initial diagnostics such as electrocardiograms (ECG).^6^ ^,^ ^7^

Despite the volume and acuity of patients treated by APPs in the ED, a relatively small proportion of APPs have received formalized postgraduate training in emergency medicine (EM), with 10% of PAs and 21% of NPs having completed such training.^2^ ^,^ ^8^ Both the American Academy of Emergency Nurse Practitioners (AAENP)^9^ and the Society of Emergency Medicine Physician Assistants (SEMPA)^10^ identify ECG interpretation as a requisite skill for APPs practicing in EM. However, no consistent approach is applied nationwide to address this lack of EM-specific training.^11^ ^,^ ^12^

Foundations of Emergency Medicine (FoEM) is a free, open-access curriculum that is widely used and validated in EM resident education.^13^ ^,^ ^14^ FoEM offers standardized, level-specific, core content that primarily targets resident physicians in EM. The FoEM ECG I course is composed of six units that review fundamental concepts in ECG interpretation using a flipped classroom approach (Appendix 1).^15^ Implementation guidelines, unit summaries, challenge ECGs, and interpretation guides are all found on the FoEM website, which may be accessed by program leaders after free registration.^16^ We sought to address a gap in training and improve APP ECG interpretation skills by implementing the FoEM ECG I course.

## METHODS

### Study Population and Design

We included APPs in this single-site study if they currently practiced at a large, urban, county hospital and were enrolled in the FoEM ECG I course during October 2020-June 2021. While enrollment in the course was required to staff higher acuity ED zones, participation in the study was voluntary. Participants reviewed unit summaries and practiced select ECGs prior to each workshop. During the workshop, APPs were divided into small groups to collaboratively review four challenge ECGs with interpretation and discussion prompts. Upon completion of small-group discussion, faculty or senior resident instructors facilitated interactive sessions with all learners, reviewing core concepts and ECG challenge answers.

Study participants completed a knowledge, attitudes, and practices (KAP) survey at the beginning and completion of the course (Appendix 2). Additionally, we obtained objective knowledge acquisition through a 15-question multiple-choice assessment administered in October 2020 (pre-intervention), December 2020 (immediate post-intervention), and June 2021 (delayed post-intervention). This study was deemed exempt by the Institutional Review Board of Emory University.

### Statistical Analysis

We described categorical variables using frequencies and percentages. Continuous and scale variables were described using medians and interquartile ranges (IQR). We evaluated the change in ECG knowledge scores between the pre-test, post-test, and delayed post-test sessions using the Friedman repeated-measures rank-order ANOVA. Ordinal self-assessment variables were evaluated using a mixed-effects ordinal logistic regression. We used mixed effects to account for multiple responses from individual study participants. Odds ratios (OR) and 95% confidence intervals (CI) are presented from the regressions. Two-tailed *P*-values ≤0.05 were considered significant. We conducted statistical analyses using R version 4 (R Core Team, Foundation for Statistical Computing, Vienna, Austria).

## RESULTS

A total of 23 APPs enrolled, with the majority identifying as female (74%) with a median age of 37 (IQR 33–40) years (Table 1). Learners were primarily family nurse practitioner (FNP) (48%), followed by physician assistrant PAs (26%) and FNP-emergency nurse practitionerENPs (22%). They reported a median of five years of postgraduate experience in EM (IQR 3–6), and a small proportion reported completing formalized postgraduate training in EM (13%).

**Table 1.** Demographic characteristics of advanced practice providers enrolled in the Foundations of Emergency Medicine ECG I course, October 2020–June 2021.

Characteristic	Value N = 23
Age, median (IQR)*	37 (33–40)
Gender, n (%)	
Female	17 (74%)
Male	6 (26%)
Certification, n (%)	
AGNP**	1 (4%)
FNP^α^	11 (48%)
FNP-ENP^β^	5 (22%)
PA^¥^	6 (26%)
Postgraduate experience, median (IQR)	5 (3–6)
Completed emergency medicine postgraduate training program, n (%)	3 (13%)

^*^ *IQR*, interquartile range; ^**^ *AGNP*, adult gerontology nurse practitioner; ^α^ *FNP*, family nurse practitioner; ^β^ *FNP-ENP*, family nurse practitioner - emergency nurse practitioner; ^¥^ *PA*, physician assistant.

**Figure 1.**
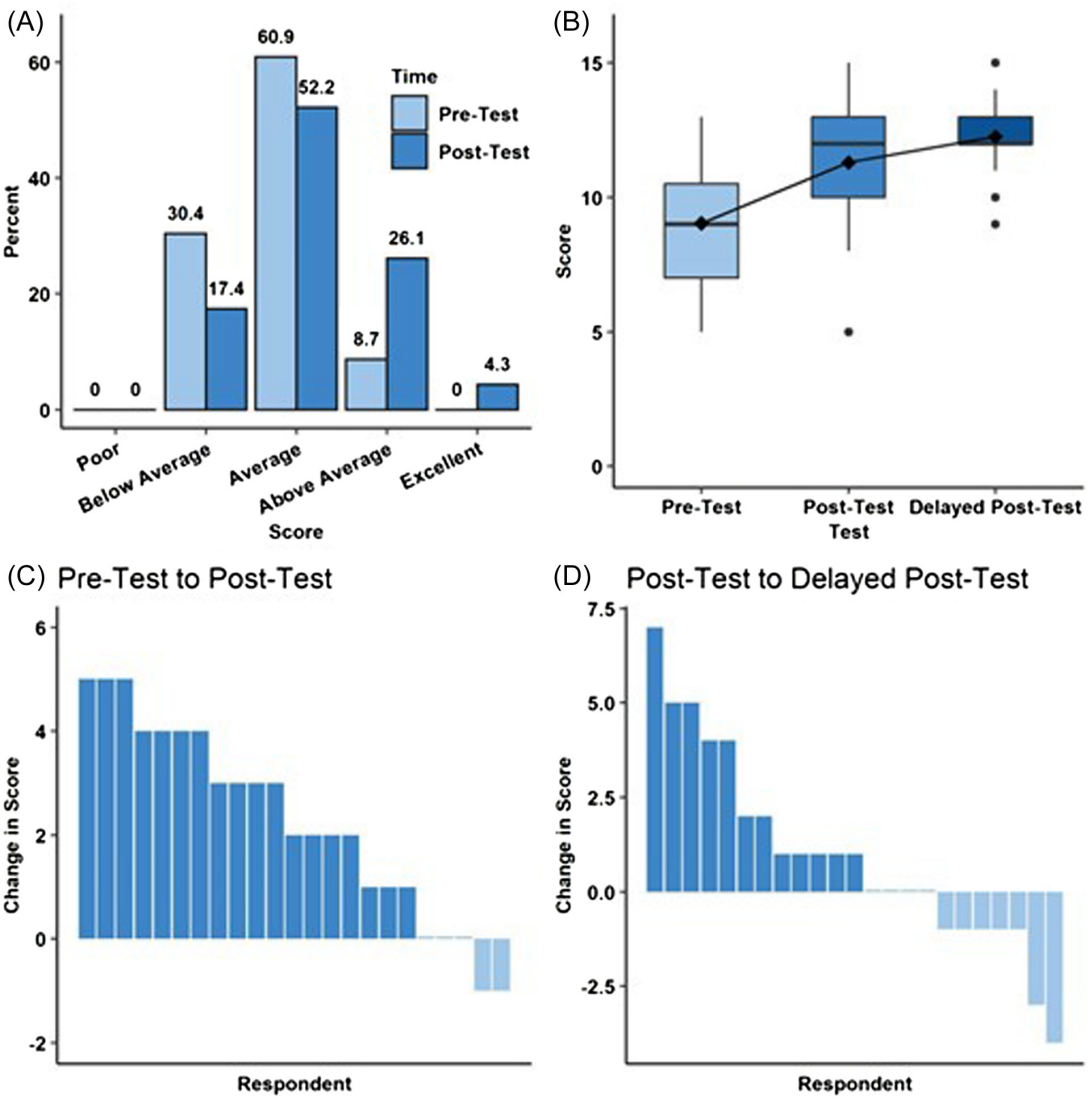
Advanced practice provider perceived and objective knowledge acquisition before and after foundations of emergency medicine (FoEM) Electrocardiogram Gram (ECG) I course. A) Self-assessment of respondents’ understanding of ECG interpretation as a function of time. B) Boxplot depicting knowledge test scores as a function of time. C) Waterfall plot depicting the change in knowledge test scores from pre-test to post-test for each individual respondent. D) Waterfall plot depicting the change in knowledge test scores from post-test to delayed post-test for each individual respondent.

Self-assessed confidence of ECG interpretation was higher in the post-test assessment compared to the pre-test assessment; however, the difference was not significant (odds ratio [OR] 2.94 (95% confidence intervalCI 0.94–9.1), *P* = 0.06) (Figure 1A). In contrast, the objective knowledge assessments (Figure 1B-D) indicate that ECG interpretation improved (*P* < 0.001). Post-hoc tests indicated that post-test scores (median 12/15, IQR10–13) were significantly greater than pre-test scores (median 9/15, IQR 7–11; *P* < 0.001). Delayed post-test scores (median 12/15, IQR 12–13) did not differ from post-test scores (*P* = 0.30) indicating that the improved understanding was largely maintained over time.

On the KAP survey, APPs reported improved confidence in detecting an ST-segment elevation myocardial infarction (STEMI) on ECG (*P* = 0.01) (Appendix 3). No overall change was noted in confidence to interpret a life-threatening arrhythmia (*P* = 0.27). Participants were no more or less likely to approach an attending physician for help regarding an uncertain ECG before and after the ECG curriculum (*P* = 0.16). With respect to participants’ view of the course, 21 (91%) reported being satisfied or highly satisfied; 9 (39%) satisfied; and 12 (52%) highly satisfied. Only one participant (4%) was neutral and one (4%) was unsatisfied. Twenty-two participants (96%) believed that the course was taught at the correct level of difficulty: 13 (57%) strongly agreed; and 9 (39%) agreed. Only one participant (4%) was neutral. No respondent disagreed.

## DISCUSSION

The FoEM ECG I curriculum was administered to APP learners and evaluated using a pre- and post-intervention, self-reported KAP survey in conjunction with an objective measure of knowledge acquisition. There was an improvement in objective knowledge and retention, a trend toward improved confidence in ECG interpretation, and a significant improvement in STEMI identification. Despite these improvements, there was no change in the APPs’ likelihood of reaching out to physicians for assistance with ECG interpretation.

A unique advantage of our study is its demonstration of measurable improvement in clinically relevant ECG interpretation. While this is encouraging with respect to the ease and efficiency of the course, what is more impressive is the retention of knowledge over time. The APPs commonly work in triage and lower acuity areas and are often the first practitioners to evaluate patients in the ED.^6^ ^,^ ^7^ To detect many life-threatening illnesses, prompt ECG acquisition and interpretation is essential. Thus, APPs should be able to interpret ECGs when a physician is not immediately available, as may be the case in some practices.^17^ Despite the importance of ECG interpretation, APPs often find this clinical skill challenging, with one study demonstrating 50% proficiency of ECG interpretation among graduating PAs, a metric commensurate with the baseline competency demonstrated in our cohort.^18^ In this study, we were able to increase and maintain competency at 80%, underscoring the value added by the FoEM ECG I course. Despite this increased knowledge, APPs were just as likely to reach out to a physician for ECG interpretation guidance, showing that the course did not decrease reliance on physician knowledge and judgment.

Our study demonstrated that APPs, despite showing increased knowledge following the course, only gained confidence in identifying STEMIs on ECG, but did not improve confidence in other domains of ECG interpretation following the FoEM ECG I course. This represents a mismatch in perceived and actual ECG interpretation competence. This may reflect limited individual time spent on each module and/or modules focused on more specific ECG pathologies. Further studies may evaluate whether confidence may be improved with continued training and exposure to more diverse ECG findings.

While implementing the course and showing it was effective from a knowledge, attitudes, and behaviors standpoint is of paramount importance, an educational program must also be well-received by the learner. Our study demonstrated very high levels of satisfaction with the course among our APPs, along with APPs reporting that the concepts taught were appropriate for their level of training. This translates into more engagement and knowledge acquisition and retention in the curriculum, in fitting with prior studies looking at APPs and case-based education.^19^

## LIMITATIONS

Our study limitations included a small sample size with a relatively homogeneous study population (eg, primarily NPs, all practicing at a single county hospital). Additionally, our study cohort did not reflect the roughly 50/50 distribution of NPs and PAs practicing in acute care settings, with our group only having 26% PA representation. Finally, our study did not include a control group which did not receive training thereby allowing for the possibility of test/retest effects.

## CONCLUSION

Formalized postgraduate ECG interpretation training for APPs in EM is at best inconsistent, yet both SEMPA and AAENP list ECG interpretation as a necessary skill for practicing in EM.^9^ ^,^ ^10^ In response to this, we implemented the FoEM ECG I course and found that it was easy to implement, led to improved ECG knowledge and confidence in ECG interpretation, and was well received by the APP group. These results may inform the use of this free, structured ECG curriculum at both academic and community-based programs that support continuing education for APPs. Future studies should investigate the impact of increased sample sizes, more variable practice locations and departmental designs, and a higher proportion of PAs, all of which would serve to make the data more reflective of the APP population as a whole and, therefore, more generalizable.

## Supplementary Information




