# Supplementary material for: Bridging the Gap: Evaluation of an Electrocardiogram Curriculum for Advanced Practice Clinicians
Source: West J Emerg Med. 2024 Feb 9;25(2):155–9. doi: 10.5811/westjem.18085 (PMC11000549; doi:10.5811/westjem.18085)
Supplement: Supplementary file 1 [file wjem-25-155-s001.docx]

**Appendix 1.** Foundations of Emergency Medicine ECG I curriculum outline.

| Unit | Unit summary |
| --- | --- |
| Unit I | How to read an ECG  Approach to ischemia: STEMIs |
| Unit II | Approach to ischemia: ischemia mimics |
| Unit III | Approach to syncope |
| Unit IV | Approach to bradyarrhythmias |
| Unit V | Approach to tachyarrhythmias: narrow complex |
| Univ VI | Approach to tachyarrhythmias: wide complex |

*ECG*, electrocardiogram; *STEMI*, ST-elevation myocardial infarction.

**Appendix 2.** APP foundations EKG course survey

Please see separate PDF file.

**Appendix 3.** Advanced practice provider self-reported ECG knowledge, attitudes, and practices before and after Foundations of Emergency Medicine ECG I course.

| Self-assessment | Pre-test  N=23  n (%) | Post-test  N=23  n (%) | OR (95% CI) | *P*-value |
| --- | --- | --- | --- | --- |
| Confidence independently interpreting STEMI |  |  | 3.12 (1.20-7.62) | *P*= 0.01 |
| Not confident | 2 (9) | 0 (0) |  |  |
| Somewhat confident | 11 (48) | 8 (35) |  |  |
| Confident | 9 (39) | 10 (44) |  |  |
| Very confident | 1 (4) | 5 (22) |  |  |
| Confidence independently interpreting life-threatening arrhythmia |  |  | 1.69 (0.67-4.27) | *P*= 0.27 |
| Not confident | 4 (17) | 3 (13) |  |  |
| Somewhat confident | 14 (61) | 12 (52) |  |  |
| Confident | 3 (13) | 5 (22) |  |  |
| Very confident | 2 (9) | 3 (13) |  |  |
| Likelihood of approaching attending for help regarding uncertain ECG |  |  | 0.30 (0.06-1.62) | *P*= 0.16 |
| Very unlikely | 0 (0) | 0 (0) |  |  |
| Unlikely | 0 (0) | 0 (0) |  |  |
| Likely | 1 (4) | 3 (13) |  |  |
| Very likely | 22 (96) | 20 (87) |  |  |

*STEMI*, ST-elevation myocardial infarction; *ECG*, electrocardiogram.
